# Efficient alignment-free DNA barcode analytics

**DOI:** 10.1186/1471-2105-10-S14-S9

**Published:** 2009-11-10

**Authors:** Pavel Kuksa, Vladimir Pavlovic

**Affiliations:** 1 Department of Computer Science, Rutgers University, Piscataway, NJ 08854, USA

## Abstract

**Background:**

In this work we consider barcode DNA analysis problems and address them using alternative, *alignment-free *methods and representations which model sequences as collections of short sequence fragments (features). The methods use fixed-length representations (spectrum) for barcode sequences to measure similarities or dissimilarities between sequences coming from the same or different species. The spectrum-based representation not only allows for accurate and computationally efficient species classification, but also opens possibility for accurate clustering analysis of putative species barcodes and identification of critical within-barcode loci distinguishing barcodes of different sample groups.

**Results:**

New alignment-free methods provide highly accurate and fast DNA barcode-based identification and classification of species with substantial improvements in accuracy and speed over state-of-the-art barcode analysis methods. We evaluate our methods on problems of species classification and identification using barcodes, important and relevant analytical tasks in many practical applications (adverse species movement monitoring, sampling surveys for unknown or pathogenic species identification, biodiversity assessment, etc.) On several benchmark barcode datasets, including ACG, Astraptes, Hesperiidae, Fish larvae, and Birds of North America, proposed alignment-free methods considerably improve prediction accuracy compared to prior results. We also observe significant running time improvements over the state-of-the-art methods.

**Conclusion:**

Our results show that newly developed alignment-free methods for DNA barcoding can efficiently and with high accuracy identify specimens by examining only few barcode features, resulting in increased scalability and interpretability of current computational approaches to barcoding.

## Background

Identification of living species is one of the pressing tasks in science and technology today, prompted by our need to understand the natural biodiversity and its increasing interaction with the human society.

However, development of comprehensive species identification strategies is impeded by the enormous biodiversity of life on Earth. Traditional morphological identification of species is difficult, requires expertise of highly trained taxonomists, and takes up enormous amounts of time. Species identification methods based on molecular diagnostic technologies, including PCR, are limited in the number of species they can identify and lack standardization of technologies or are susceptible to tissue conditions. DNA barcoding has been recently introduced as a taxonomic tool for characterizing species using fragments of a DNA sequence from standard gene regions, such as the mitochondrial DNA (mtDNA) [1]. These relatively short sequences (about 650 symbols in the case of mtDNA) are used as markers for discerning taxonomical identities of specimens using the process of mtDNA extraction, fragment amplification, sequencing and database lookup [2]. A critical property of this particular region is its monophyletic association: the content of mtDNA is often preserved within a species and shows greater divergence between than within species (sometimes 10× or more when sister species are excluded) [3]. In particular, a region corresponding to *c oxidase subunit 1 *or cox1 gene is often used as a critical barcoding marker [1] that exhibits such properties.

Barcoding has shown great promise in practice. DNA barcodes can offer increased adaptability, robustness, and predictive value for rapid and accurate identification of species. For instance, barcoding analysis can result in improved correct placement of previously unknown species or increased resolution of specimens [4], identification of fish products with high accuracy [5], substitutes in fish species for human consumption [6] or marketing of endangered specimens [7]. DNA barcoding has been applied with great initial success to identification across the spectrum of living species, from algea [8], fungi [8], bacteria [9], to plants [10-12], spiders [13], fish [14], birds [1], and rats [15].

Most current barcoding computational methods leverage established modeling approaches from molecular phylogenetic analysis. Traditional barcoding methods, c.f., [1,16], are essentially tree-based phylogenetic approaches where identification decisions are made using a-priory threshold on the tree-induced distances. Choosing an optimal threshold is a challenging task, affected by variable relationship between the species morphology and the cox1 content similarity. More recently, sophisticated Bayesian and decision theory approaches [17,18] have been proposed that attempt to address this problem in a more systematic manner. Traditional phylogenetic methods are also sensitive to the choice of the sequence similarity metrics and the presence of exogenous variations in the sequence (such as those caused by bacterial cosegregation). Moreover, methods of molecular phylogeny are not inherently aimed at the task of sequence delineation, rather the study of relationships at different points in evolutionary history. As a consequence, they can also sometimes exhibit high computational complexity, justified for the complex analysis task but often unnecessary when the goal is e.g., species identification.

More recently, methods that more directly tackle the problem of barcode-based identifications have emerged. Some of these methods, such as [16] use the tools of generic but widely available and highly computationally optimized biological sequence comparison (BLAST or PSI-BLAST). Approach such as [19] even more immediately focuses on the prediction problem. However, a number of challenges remain to be addressed, including the accuracy of identification [16,18,20,21], as well as the efficiency and scalability of computational methods.

In this study we investigate *alignment-free kernel *methods for the DNA barcoding. *Kernel-based classification *has demonstrated strong performance in many related tasks of biological sequence analysis, such as protein classification and remote homology detection [22-24]. In the process, a number of kernel types or *similarity measures *between sequences have been proposed, including kernels derived from probabilistic models [25], *k*-mer string kernels [22,23], and weighted-decomposition kernels [26]. In this work we focus on *k*-mer string kernels, and in particular the spectrum/mismatch kernel methods. In our approach, species identification is performed by first transforming variable-length sequences into fixed-length representations (string spectra) and then classifying resulting spectral representations into one of many established species classes using state-of-the-art classification algorithm (e.g. nearest neighbor or Support Vector Machine (SVM) classifiers [27,28]). As a result, the alignment free kernel-based species identification in our study demonstrates both high accuracy, improved speed and classification performance compared to previously employed DNA barcoding identification methods.

## Methods

In this section we discuss alignment-free analytics that we propose to use for accurate and efficient multi-class classification and identification of barcode sequences.

### The spectrum kernel methods

Varying sequence length as well as the warping processes within sequences (insertions/deletions) typically preclude direct application of efficient computational models and algorithms designed for data in Euclidean spaces. The spectrum kernel methods [29,30] resolve this problem using fixed-length representations of arbitrary long sequences. These representations or *features *describe the statistics of short substrings of length *k*, also known as *k-mers*, contained in the original sequence. Such representations are both efficient to compute and informative for the tasks of sequence analysis.

Consider a sequence *X *of length *n *represented as a string of symbols (*x*_1_, *x*_2_,..., *x*_*n*_) from some alphabet Σ, *x*_*i *_∈ Σ. In the case of DNA sequences this alphabet consists of the set of the four DNA bases, {A, C, T, G}. Spectrum methods construct a fixed-length feature vector Φ(*X*) from this arbitrary long sequence by counting the frequencies of occurrence of all *k*-mers *x*_*i*_, *x*_*i*+1_,..., *x*_*i*+*k*-1 _in *X*. This feature, the histogram of *k*-mers in *X*, is commonly referred to as the sequence *spectrum*. The spectrum's domain has the dimension |Σ|^*k *^corresponding to the total number of all possible fragments of length *k *and, as a result, induces a fixed length representation.

**Figure 1:**
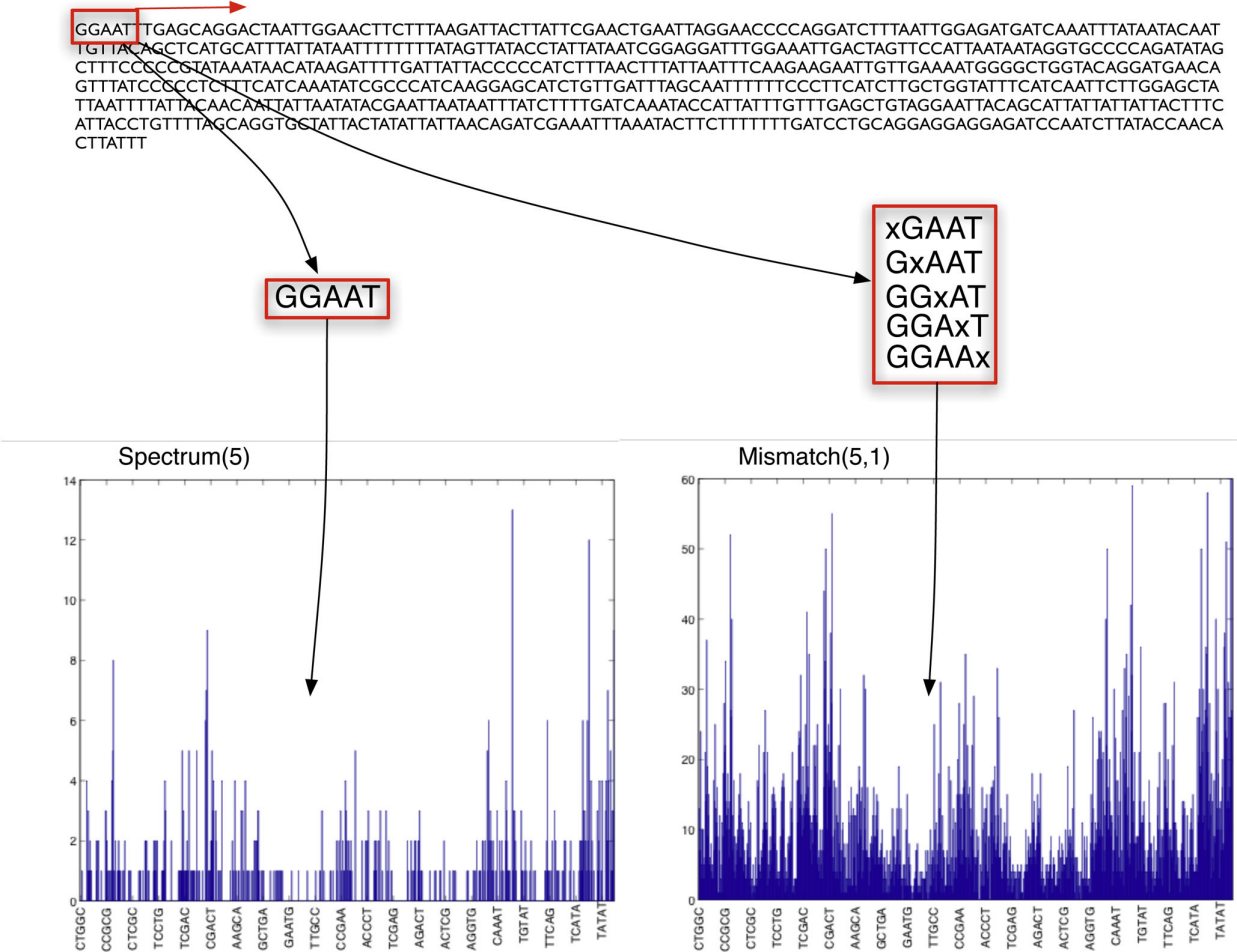
**Illustration of spectrum and mismatch features**.

This concept is illustrated in Figure 1. A sequence from the Astraptes set is represented as the histogram of frequencies with which 5-long fragments (5-mers) occur in that sequence. In the case of 5-mers there are Σ^*k *^= 4^5 ^= 1024 such possible fragments, some of which are identified on the horizontal axes of the count plots in Figure 1. For instance, the fragment "CCGCG" occurs three times. Hence, the Astraptes sequence is mapped to a 1024-dimensional fixed-length representation. This representation will be subsequently used to judge similarities and dissimilarities between pairs of sequences coming from the same or different species.

In practice the spectrum mapping will produce sparse feature vectors of counts when either *k *is long or the sequences are short. On average and assuming a random sequence generation process, for a sequence of length *n *each feature will appear *n*/|Σ|^*k *^times. While the use of larger *k *is preferred to yield higher specificity of features, it inadvertently can lead to representations or feature spaces that are too high dimensional and produce low similarity even between sequences in the same class (species). As a consequence, it is often necessary to increase the "density" of these features to allow sufficient within-class sensitivity while maintaining the specificity across classes.

Increasing density for a fixed *k*-mer length can alternatively be viewed as the process of inexact sequence matching. The *mismatch *kernel method [29] accomplishes this task using the following general *mismatch*(*k*, *m*) |Σ|^*k*^-dimensional representation of sequence *X*:

(1)

where *I*_*m*_(*α*, *γ*) = 1 if *α *∈ *N *(*γ*, *m*) and *N*(*γ*, *m*) denotes the set of contiguous substrings of length *k *that differ from *γ *in at most *m *positions. In other words, in addition to counting all *k*-mers *α *present in sequence *X*, one also adds counts of *k*-mers that differ in at most *m *symbols from each *α*. This process is illustrated in Figure 1 where 5-mer "GGAAT" is mapped to a set of ·(|Σ|^*m *^- 1) + 1 = 5 × 3 + 1 = 16 similar *k*-mers, at most one symbol (*m *= 1) different from "GGAAT". The induced feature vector Φ^*k*, *m*^(*X*) has the same dimension as the regular spectrum feature Φ(*X*), but is "denser". The choice of the maximum number of the mismatches (*m*) allowed between any two particular *k*-mers typically depends on whether sequences are relatively similar (e.g. closely related families, *m *is small) or are far apart (e.g. remote homologs, large values of *m *may be needed). The *exact *spectrum kernel is a particular case of the mismatch kernel and can be obtained from Eq. 1 by setting the number of mismatches *m *to zero (this will result in counting only exact matches between *k*-mers). Both mismatch and exact spectrum methods measure similarity of sequences by comparing the fixed-length features Φ^*k*, *m*^of those sequences without performing any sequence alignment. As we discuss in the next section, the computational cost of evaluating this similarity is *linear *in the length of the sequences, compared to *quadratic *complexity required by alignment-based methods (e.g. Smith-Waterman) for similarity evaluation. This leads to a potentially important advantage for these methods when applied to large DNA barcode sets, which we demonstrate empirically in our Results.

### Alignment-free algorithms

Both mismatch and spectrum methods typically evaluate similarity *K*(*X*, *Y*) of a pair of sequences by computing the dot-product between their corresponding feature vectors (Eq. 1):

(2)

Direct evaluation of the dot-product above for similarity computation results in costly *O*(|Σ|^*k*^*n*) complexity. To efficiently evaluate the dot-product, we first note that in Eq. 2 the product *I*_*m*_(*α*, *γ*)*I*_*m*_(*β*, *γ*) is non-zero (i.e. contributes to the total similarity/kernel value) only if *γ *is the neighbor for both *α *and *β*. We then write the dot-product (Eq. 2) as follows:

(3)

where *I*_*k*, *m*_(*α*, *β*) is the number of *k*-mers *γ *shared by *α *and *β*. We observe that the number of shared *k*-mers *I*_*k*, *m*_(*α*, *β*) depends on the Hamming distance (i.e., the number of differences, in symbols, between the strings) *d*(*α*, *β*) between *α *and *β *for a fixed alphabet Σ, the length of the *k*-mer *k*, and the number of mismatches *m *(i.e. *I*_*k*, *m*_(*α*, *β*) can only have a fixed set of values with each value corresponding to a particular Hamming distance). Since the maximum Hamming distance that will result in the non-zero *I*_*k*, *m*_(*α*, *β*) is 2*m*, the dot-product in Eq. 3 reduces to computing the number of pairs (*α*, *β*), *α *∈ *X*, *β *∈ *Y*, for each of possible Hamming distances from 0 to 2*m*:

(4)

As we show in [31], the mismatch/spectrum similarity measure in the form as in Eq. 4 can be efficiently computed in *O*(*c*_*k*, *m*_*n*) time, where *c*_*k*, *m *_is a constant that depends only on the *k*-mer length and the maximum number of allowed differences *m *but not on the sequence length *n*. In the case of the exact spectrum method, the complexity is *O*(*kn*), i.e. is linear in both the sequence length *n *and the *k*-mer length *k*. It is also important to note that we typically need to evaluate this similarity for a set of *N *sequences (e.g., DNA barcode samples). Instead of evaluating similarity for every pair of *N *sequences, a task proportional to *N*^2^, in [31] we also show that this can be accomplished in the time linear in *N*. Hence, the overall complexity of evaluating the mismatch(k, m) similarity on a set of *N *sequences of maximal length *n *is *O*(*c*_*k*, *m*_*nN*). This results in significant computational savings (speedup) when it is necessary to compute similarity among a large number of sequences, as may be the case with DNA barcodes.

### Prediction models

Given the similarity kernel for any pair of sequences, one can consider several predictive tasks. One such task is the classification of new sequence samples into one of the previously seen classes. In the context of DNA barcoding, this task can be interpreted as either the classification of a barcode sample into one of the known species or the verification task of resolving whether the sample belongs to a particular species or not. We first consider the latter (verification) task and then generalize it to the full classification task. A very general class of predictive models that relies on the similarity metric induced by the kernel *K *computes the matching score between the query sequence *X *and the previously seen sequences {*X*_1_,..., *X*_*N*_} whose class assignments {*y*_1_,..., *y*_*N*_} are known. The score is formed as

(5)

The sign of this score then typically indicates whether the query *X *belongs to a particular class, *f*(*X*) > 0, or not. The weights *w*_*i *_are set in a training procedure prior to making predictions using a variety of available "learning" algorithms that attempt to optimize the predictive performance of this model. This verification model can also be generalized to the classification setting, where the sample is to be classified in one of *M *possible classes. In that case one can construct the predictive model for each class, *f*_*m*_(*X*) = Σ_*i*_*w*_*m*, *i*_*K*(*X*, *X*_*i*_), and make the final prediction by finding the class with the maximum score, y* = arg max_*m *_*f*_*m*_(*X*).

In this work we consider two classes of algorithms that have generally shown state-of-the-art performance on prediction tasks. One is the simple Nearest Neighbor classifier. In that setting *w*_*m*, *i *_is non-zero, i.e. *w*_*m*, *i *_= 1, only for the sequence *X*_*i *_(of class *y*_*i *_= *m*) which is "closest", or most similar, to the query sequence *X*. Nearest neighbor classifiers are simple and have appealing (asymptotic) theoretical properties.

The second class of learning algorithms used in this work is the well-known Support Vector Machine [28]. In the view of the model above the SVM selects an optimal subset of training sequences *X*_*i *_(the so-called support vectors) and sets their weights to maximize the models predictive accuracy. In our work we use the "one-vs-rest" SVM learning approach described in [32].

## Results and discussion

To demonstrate the utility of the alignment-free sequence representation for DNA barcode analytics we primarily focus on the task of species identification. The identification or classification task is one of the relevant analytical problems considered so far in DNA barcoding [16,18,20,21]. In this section we show that the spectrum-based, alignment-free representation possesses several interesting properties, among them the high accuracy of the sample-to-species assignments as well as the computational efficiency. Moreover, the spectral representations offer interesting insights into which sequence markers/features within the standard barcode region (e.g. cox1) serve as the most important discriminants among the sets of species. This result has further implication on computational efficiency but may also facilitate further taxonomical studies. We perform the barcode-based species classification experiments using several benchmark barcode datasets from various barcode collecting campaigns for mammals, fish, birds, lepidoptera, etc. In particular, we use seven data sets of DNA barcodes including Astraptes (12 species), Hesperiidae (364 species), Bats of Guyana (96 species), Fish of Australia (211 species), Birds of North America (656 species), ACG (573 species), and Fish larvae (7 species). Astraptes, Hesperiidae, Bats of Guyana, Birds of North America, and Fish of Australia were compiled from the BOLD [33] project. ACG set was published as a part of [34]. The Fish larvae set appeared in [16]. Table 1 summarizes details of these datasets.

**Table 1:** Barcode datasets

**Dataset**	**# species**	**# barcodes**
ACG	573	4267
Hesperiidae	364	2185
Astraptes	12	465
Bats of Guyana	96	840
Birds of North America	656	2589
Fish of Australia	211	754
Fish larvae	7	35

Using these datasets, we consider barcode class prediction problem as a multi-class classification problem described in the Methods section. For the SVM prediction approach, we use one-vs-rest setting to perform the multi-class classification using binary predictors for each class. We evaluate alignment-free similarity of DNA barcodes using the spectrum/mismatch representations of Section 'The spectrum kernel methods' and contrast it to several standard similarity metrics employed for biological sequences and DNA barcodes in particular. In all experiments, we normalize the similarity/kernel values *K*(*X*, *Y*) using *K'*(*X*, *Y*) = *K*(*X*, *Y*)/ to remove the dependency between the kernel value and the sequence length. To perform our experiments, we use an existing SVM implementation from a standard machine learning package SPIDER [35] with default parameters. For the spectrum/mismatch kernel, we use mismatch(5,1) (*k *= 5, and *m *= 1) and spectrum-10 (*k *= 10) kernels. To facilitate experiments on large datasets, we use the kernel computation algorithms proposed by Kuksa et al. in [31]. The data and source code used in our experiments are available at the supplementary website [36].

In the following, we first present results on multi-class species identification problem using alignment-free methods. We then focus on the analysis of within-barcode markers and show the impact of the marker selection on the identification accuracy. To illustrate the ability of the similarity metric to reduce the within-species dispersion while maintaining separability of different species we use clustering analysis in the set of experiments following the marker study. Finally, we provide empirical running time analysis of our proposed approach and contrast it with some state-of-the-art methods.

### Species identification

In the species identification experiments, we use the nearest neighbor classifier and the SVM to predict class assignments for query barcodes based on similarity scores computed using alignment-based (Smith-Waterman, Kimura, Hamming) and *alignment-free *methods (spectrum and mismatch kernels). To discern the predictive ability of different methods we consider a cross-validation setting in which the species data is randomly split into ten disjoint subsets. Nine of the subsets are used to estimate the prediction models which are subsequently evaluated on the remaining set, in a repeated fashion. We report results averaged over the ten folds and show the accuracy variation of each method.

#### Nearest neighbor approach

Classification performance for the nearest neighbor approach using *alignment-free *kernel methods is summarized in Table 2 where we compare cross-validation error rates of the resulting classifiers on the benchmark barcode datasets. We also report results obtained by running PSI-BLAST search with default parameters on these datasets. The results indicate that the alignment-free spectral method generally shows the highest classification accuracy. Compared to PSI-BLAST search, the mismatch similarity exhibits similar results, typically inferior to those of the spectral similarity. In Table 3 we show classification performance of the spectrum method for different values of *k *(*k*-mer length). The error rates are shown for the nearest neighbor (1-NN) and for 3-NN and 5-NN classifiers. We observe that the nearest neighbor classifier displays the lowest error rates compared to classifiers that use 3 or 5 nearest neighbors for prediction. As we can see from the table, the spectrum method is relatively robust to the choice of the *k*-mer length, with values of *k *= 8 - 15 resulting in the highest classification accuracy. We also note that the experiments show that the smaller *k *are satisfactory and increasing *k *much does not increase accuracy significantly; smaller values of the *k*-mer length also have lower computational complexity compared to the larger values of *k*.

**Table 2:** Nearest neighbor, 10-fold cross-validation error (%)

**Dataset**	**PSI-BLAST**	**spectrum**	**mismatch**
ACG	3.07 ± 0.68	2.49 ± 0.87	3.63 ± 0.65
Hesperiidae	4.62 ± 0.97	3.57 ± 1.08	4.38 ± 1.49
Astraptes	13.82 ± 4.42	1.07 ± 1.81	1.50 ± 1.99
Bats	1.63 ± 1.22	1.63 ± 1.22	1.73 ± 2.01
Birds	7.46 ± 1.90	6.22 ± 1.50	7.29 ± 0.96
Fish Australia	5.62 ± 3.31	5.5 ± 3.27	5.29 ± 3.34
Fish larvae	2.86	2.86	5.71

**Table 3:** Classification performance of the *k*-spectrum method using nearest neighbor

**dataset**	**k = 3**	**k = 5**	**k = 8**	**k = 10**	**k = 15**
1-NN (nearest neighbor)					

ACG	4.24 ± 0.90	3.19 ± 0.93	2.58 ± 0.94	2.49 ± 0.87	2.35 ± 0.83
Hesperiidae	5.32 ± 1.33	4.21 ± 1.18	3.66 ± 1.07	3.57 ± 1.08	3.39 ± 0.93
Astraptes	1.91 ± 1.87	1.90 ± 2.08	1.48 ± 1.75	1.07 ± 1.81	1.07 ± 1.81
Bats of Guyana	1.87 ± 1.36	1.63 ± 1.22	1.63 ± 1.22	1.63 ± 1.22	1.63 ± 1.22
Birds	7.77 ± 1.26	6.68 ± 1.22	6.42 ± 1.34	6.22 ± 1.50	6.13 ± 1.65
Fish Australia	5.47 ± 3.26	5.35 ± 3.36	5.35 ± 3.36	5.50 ± 3.27	5.50 ± 3.27
Fish larvae	8.57	5.71	2.86	2.86	2.86

3-NN					

ACG	10.20 ± 1.31	8.98 ± 1.23	8.54 ± 1.11	8.67 ± 1.33	8.63 ± 1.21
Hesperiidae	15.55 ± 1.25	14.30 ± 1.46	14.22 ± 1.50	14.40 ± 1.85	14.30 ± 2.00
Astraptes	2.78 ± 2.49	2.36 ± 2.16	2.36 ± 2.16	2.15 ± 2.29	1.70 ± 1.96
Bats of Guyana	3.77 ± 1.68	4.46 ± 2.06	4.35 ± 2.01	4.46 ± 2.06	4.46 ± 2.06
Birds	20.23 ± 2.64	19.58 ± 2.48	18.88 ± 2.29	18.99 ± 2.22	18.37 ± 2.07
Fish Australia	12.44 ± 5.67	12.32 ± 5.68	12.31 ± 5.38	12.42 ± 5.46	11.93 ± 5.06
Fish larvae	14.29	14.29	11.43	11.43	11.43

5-NN					

ACG	13.41 ± 2.00	12.42 ± 1.40	11.49 ± 1.28	11.49 ± 1.25	11.28 ± 1.20
Hesperiidae	19.70 ± 1.71	19.64 ± 2.62	18.63 ± 2.21	18.54 ± 2.17	18.22 ± 2.17
Astraptes	3.43 ± 3.09	3.01 ± 2.76	2.14 ± 1.74	1.70 ± 1.96	1.06 ± 1.12
Bats of Guyana	6.09 ± 3.01	5.85 ± 3.23	5.73 ± 2.77	5.87 ± 2.94	5.61 ± 2.79
Birds	27.32 ± 2.50	26.26 ± 2.17	26.49 ± 2.11	26.42 ± 2.44	26.10 ± 2.36
Fish Australia	19.40 ± 5.91	18.85 ± 6.15	19.28 ± 5.28	18.36 ± 5.04	18.81 ± 4.64
Fish larvae	22.86	22.86	22.86	22.86	22.86

In Table 4, we show classification results contrasting the spectral method with nearest neighbor predictors based on more traditional, alignment-based similarity measures. We consider methods based on both global and local alignments. The global alignment on the set of sequences is obtained using the Needleman-Wunsch algorithm with *NUC44 *scoring matrix and gap opening/extending penalties set to 8. These settings result in multiple alignments largely identical to those available via BOLD [33] for the barcode sets of the publicly available projects we use in our evaluation. Given the global alignment, the sequence similarity is scored using two metrics: the Hamming distance (0/1 mismatch/match score) and the Kimura distance. For local alignment-based pairwise scores we use the Smith-Waterman scoring with the same parameters as the global alignment model. The results in Table 4 indicate that the *alignment-free *spectral similarity yields the overall most accurate species predictors. The spectral and Kimura-based distances produce comparable accuracies on three sets (Bats, Birds and Fishes of Australia). As expected, the Hamming 0/1 scoring is typically inferior to other methods as it does not include any measure of varying evolutionary pressures exhibited across different nucleotide pairs. The ACG and the Fish larvae sets are both cases where the spectral method achieves the most accurate prediction among the four contrasted scoring metrics.

**Table 4:** Nearest neighbor performance (10-fold cross-validation error, %)

**Dataset**	**Spectrum**	**Hamming**	**Kimura**	**Smith-Waterman**
ACG	**2.49 ± 0.87**	11.44 ± 1.52	5.51 ± 0.86	3.66 ± 0.66
Hesperiidae	**3.57 ± 1.08**	14.49 ± 2.36	3.81 ± 1.26	5.45 ± 1.20
Astraptes	**1.07 ± 1.81**	3.61 ± 2.77	1.71 ± 1.96	1.64 ± 1.03
Bats Guyana	**1.63 ± 1.22**	2.72 ± 1.83	**1.63 ± 1.22**	**1.63 ± 1.22**
Birds of North America	**6.22 ± 1.50**	18.38 ± 2.05	**6.02 ± 1.36**	8.20 ± 1.53
Fish Australia	**5.50 ± 3.27**	5.87 ± 4.01	**5.35 ± 3.36**	5.35 ± 3.36
Fish larvae^†^	**2.86**	11.43	8.57	5.71

^† ^leave-one-out validation error is reported

**Figure 2:**
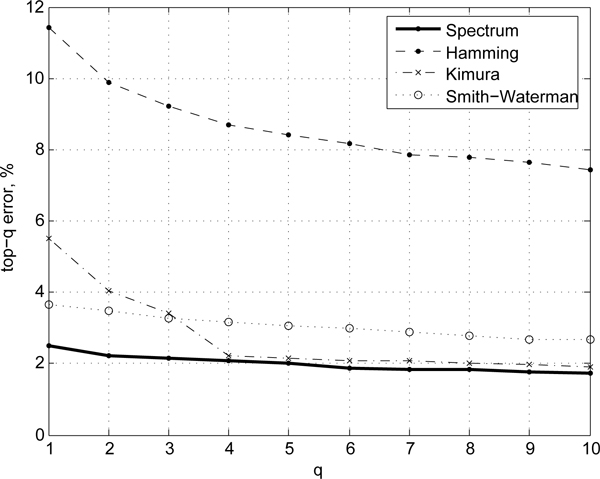
**Ranking quality**. Comparison of top-*q *error rates on ACG dataset for the spectrum (alignment-free) method and alignment-based methods.

**Figure 3:**
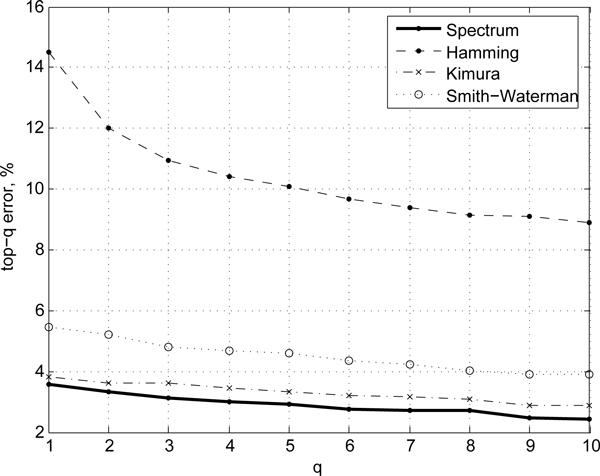
**Ranking quality**. Comparison of top-*q *error rates on Hesperiidae dataset for the spectrum (alignment-free) method and alignment-based methods.

**Figure 4:**
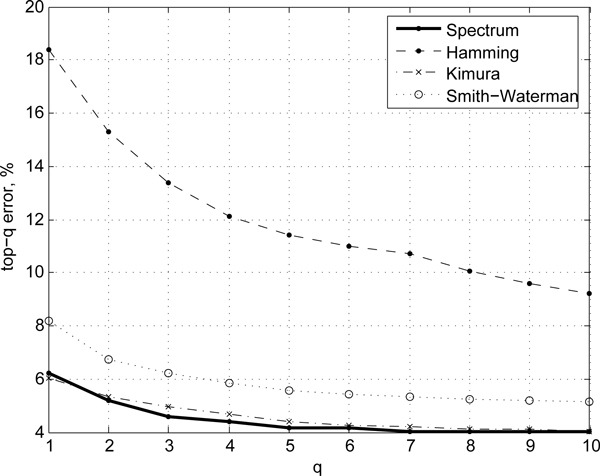
**Ranking quality**. Comparison of top-*q *error rates on Birds dataset for the spectrum (alignment-free) method and alignment-based methods.

To better assess the predictive ability of different measures we also compare the *ranking *quality of the resulting classifiers on ACG, Hesperiidae, and Birds of North America data sets in Figures 2, 3, and 4, respectively. The ranking score can be used to ascertain how closely the predictions of a model match those of the ideal case as a function of the model specificity. For instance, the top-3 error rate reports the accuracy of prediction if one assumes that correct prediction is made whenever the true species class of a sequence is anywhere among the top-3 scoring classes predicted by a model. Top-1 error rate corresponds to the standard error rate. The higher the *q*, the lower the top-*q *errors are, at the expense of the specificity of predictions. For good models/similarity measure the ranking error rate typically drops off quickly. In our evaluations we observe that the alignment-free spectrum method consistently shows lower top-*q *error rates (*n *= 1...10) compared to that of alignment-based (Kimura, Smith-Waterman, Hamming) scoring methods (Figures 2, 3, and 4), with the Kimura score approaching the spectrum for intermediate values of *n *on the sets where the two initially differ. On the ACG set, for instance, the Kimura distance-based scoring becomes comparable to the spectrum measure for *n *= 4, which suggests that further tuning of the score parameters may improve Kimura performance. However, doing so would require more complex, most likely heterogeneous, sequence models.

The above sets of experiments indicate that the alignment-free spectral measure may be well-suited for the DNA-barcode based species prediction tasks. In contrast with the alignment methods, the spectral alignment-free scores leave out the need for sometimes complex and sensitive global alignments. The benefit becomes more significant if one keeps in mind that any addition of new sequences to the set may require (global) re-alignments within a set, the step not necessary in the case of spectrum scores.

One other particularly interesting conclusion is that the spectral methods are better than or similar to the alignment-based scoring metrics even when those metrics take into account differences in evolutionary pressures across different sequence symbols, either imposed by the alignment parameters or the scoring such as that of the Kimura model. This observation should be placed in the context of our alignment-free methods that use the 0/1 scoring but within the short sequence fragments only. Using such scoring on the full sequence scale, as in the Hamming distance measure above, is bound to produce inaccurate species matches. Another important conclusion is that in the case of barcodes the mismatch alignment-free measures generally result in similarities that less accurately model the distribution of sequences within and across different species compared to the exact spectrum methods. This is in contrast with other data domains where the mismatch features have been successfully applied, such as that of the protein sequences [31]. Two factors play a role in this discrepancy. The protein sequences, from the point of our mathematical representation, live in higher-dimensional spaces due to the increased alphabet size (20 vs. 4) and hence may require a looser notion of matching. Another factor is the variability within classes (species in barcodes and, e.g., superfamilies in proteins); protein sequences with the same class typically exhibit much higher primary sequence variability than do the DNA barcodes within taxonomic groups. As a consequence, the mismatch measures may not be deemed necessary for the DNA barcoding analytics. A final comment relates to the complexity of computing the proposed measures in conjunction with their predictive performance. As we will demonstrate in Section 'Experimental running time analysis', the alignment-free spectral methods generally incur significant computational advantage over the competing alignment-based measures. This can be a deciding factor when the methods are applied to large barcode sets or in instances when new samples become available and the (global) re-alignment is not desirable.

#### SVM-based classification

SVM-based classifiers are typically regarded as state-of-the-art predictors across a wide span of modeling problems. We therefore explore the use of this class of models in the DNA barcode setting. Table 5 displays cross-validation error rate of the SVM classifier on the barcode sets in our study for the two alignment-free scoring metrics of interest. The results demonstrate that using SVM classifiers with alignment-free methods results in similar performance compared to the previously examined nearest neighbor approach. We observe slight but insignificant improvements in average error rates for the spectrum method on ACG, Hesperiidae, and Astraptes data sets, e.g. error rate reduces from 1.07 to 0.86 on Astraptes data set. These results are not unexpected, given the relatively large number of classes (species) in these settings as well as the already low error rates of the nearest neighbor predictor. At the same time, the results suggest that the spectral scoring metric for DNA barcodes appears to fairly accurately reflect the sequence diversity within and between species, and is not significantly affected (nor can be further shaped) by the choice of the predictor/classifier algorithm. We finally note that in our experiments which are not reported here we observed that other measures, which rely on global or local alignments such as the ones in Section 'Nearest neighbor approach', are similarly not affected by the choice of classification models.

**Table 5:** SVM 10-fold cross-validation error rate (%)

**Dataset**	**spectrum (k = 10)**	**mismatch**
ACG	2.32	3.48
Hesperiidae	3.25	3.36
Astraptes	0.86	1.07
Bats	1.63	1.67
Birds	5.99	7.09
Fish Australia	5.35	5.35
Fish larvae	2.86	5.71

#### Comparison with previously published results

We observe that alignment-free methods considerably improve identification accuracy compared to the previously reported results of [18,20]. For example, on *Astraptes *dataset [37], the test error rate of the multi-class SVM is only 0.86% compared to 9% in [20] or 20% in [18]. These results further signify the potential of the proposed measures as applied to the barcoding-based prediction task as well as to barcode analytics in general.

### Barcode marker selection

DNA barcodes provide full-length barcode sequences for barcode analytics. However, there are situations where only certain (few) markers within the barcode may be sufficient to accurately and rapidly perform the analytic task. Species prediction based on barcodes, and in particular for the case of a limited number of species, is one such task. The use of markers instead of the full sequence may increase the robustness of predictions by eliminating the potentially irrelevant portions of the barcodes that can also contain errors in barcode data collection. Identification of sequence markers can be also interesting from the perspective of further within-sequence loci analysis. Finally, the use of few markers can also be advantageous from the computational perspective. In this section, we evaluate the marker or feature selection performance using alignment-free methods.

In our experiments, we use the RELIEF [38] feature selection algorithm to find subsets of discriminative spectral markers. Using the *k*-mer or fragment notation we introduced previously, the markers correspond to those *k*-mers most relevant for species identification. Table 6 displays error rates of the nearest neighbor classifiers as a function of the number of selected markers.

**Table 6:** Feature selection performance using alignment-free methods (error, %)

	**# features selected**						
**Dataset**	**Full feature set (1048576 feat.)**	**4096**	**2048**	**1024**	**512**	**200**	**100**
ACG	2.49 ± 0.87	2.51 ± 0.95	2.79 ± 1.02	3.00 ± 0.96	3.17 ± 0.86	3.52 ± 0.64	4.48 ± 0.86
Hesperiidae	3.57 ± 1.08	3.53 ± 1.12	3.80 ± 1.22	4.17 ± 1.05	4.40 ± 1.15	4.81 ± 1.30	5.64 ± 1.20
Astraptes	1.07 ± 1.81	0.44 ± 0.92	0.44 ± 0.92	0.44 ± 0.92	0.44 ± 0.92	0.64 ± 1.03	1.49 ± 1.75
Bats of Guyana	1.63 ± 1.22	1.63 ± 1.22	1.63 ± 1.22	1.63 ± 1.22	1.63 ± 1.22	1.63 ± 1.22	1.63 ± 1.22
Birds	6.30 ± 1.80	6.45 ± 1.82	6.94 ± 2.08	7.13 ± 2.05	7.41 ± 1.77	9.10 ± 1.64	9.84 ± 1.99
Fish of Australia	5.50 ± 3.27	5.35 ± 3.36	5.35 ± 3.36	6.14 ± 3.50	6.80 ± 3.15	8.32 ± 2.75	9.51 ± 2.40
Fish larvae	2.86	0	0	0	0	0	0

As evident from the table, using marker selection results in the performance similar to that when full barcode sequences are used. Even when only few (about 200-500) markers are selected the alignment-free methods still result in accurate prediction and perform on-par with alignment-based methods, while being computationally more efficient, as we show in Section Experimental running time analysis. Note that the number of selected markers (e.g., 100) is the total number of marker per set of all sequences and species. The average number of markers per sequence is typically between 20% and 40% of the total number of markers (i.e., when top 500 markers are selected, each sequence is represented with about 100-200 loci, a reduction of 70% to 85% from the approximately 650 bases of the full barcode). It is also interesting to note that selecting a smaller set of markers instead of the full barcodes can sometimes lead to more robust and accurate identification. For instance, in the case of Fish larvae set the use of fewer features increases the accuracy. One reason for this may be the presence of distractors or sequencing errors that are eliminated when only the most critical markers are selected.

**Figure 5:**
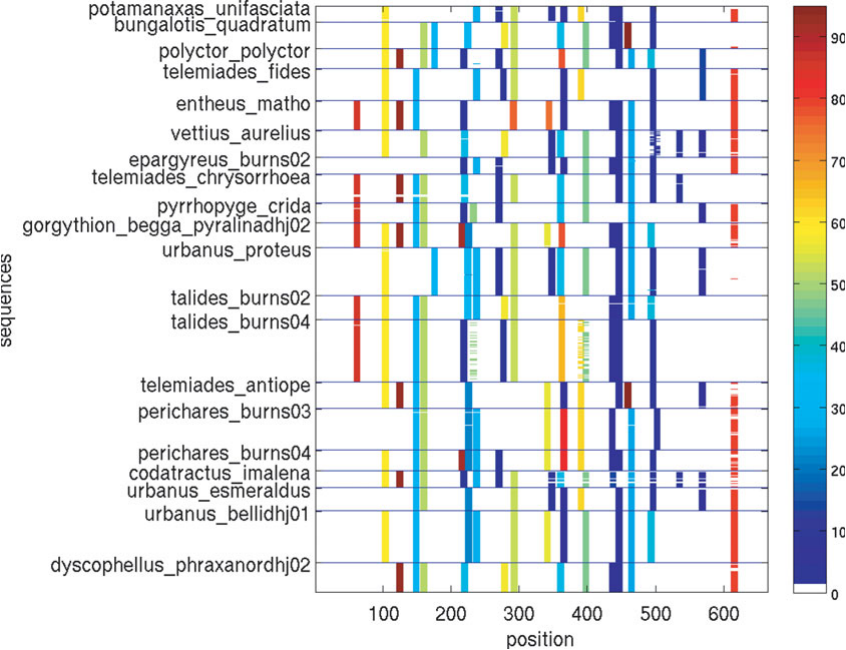
**Species sequence map for Hesperiidae data set (top 100 spectrum (*k *= 10) features)**.

**Figure 6:**
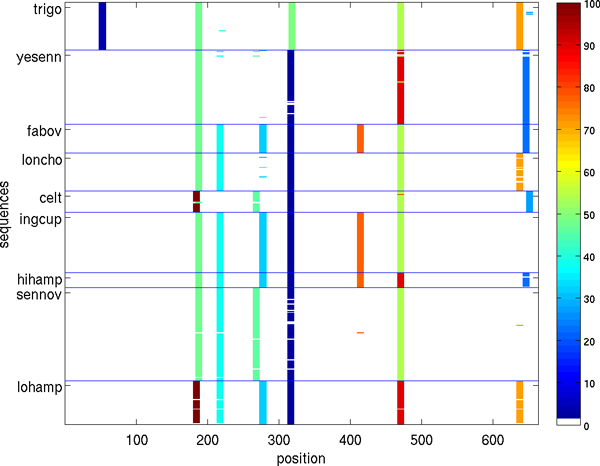
**Species sequence map for Astraptes data set (top 100 spectrum (*k *= 10) features)**.

**Figure 7:**
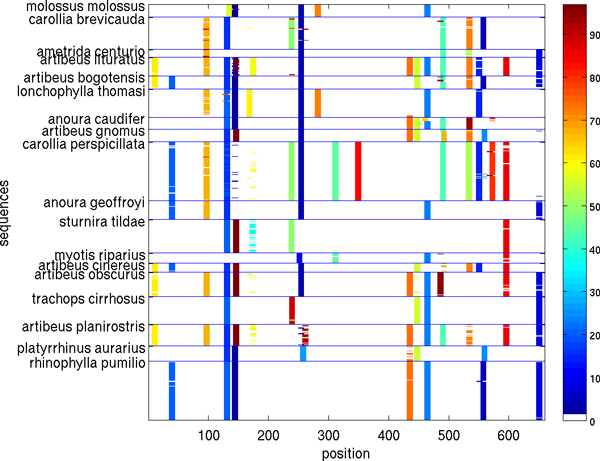
**Species sequence map for Bats of Guyana data set (top 100 spectrum (*k *= 10) features)**.

**Figure 8:**
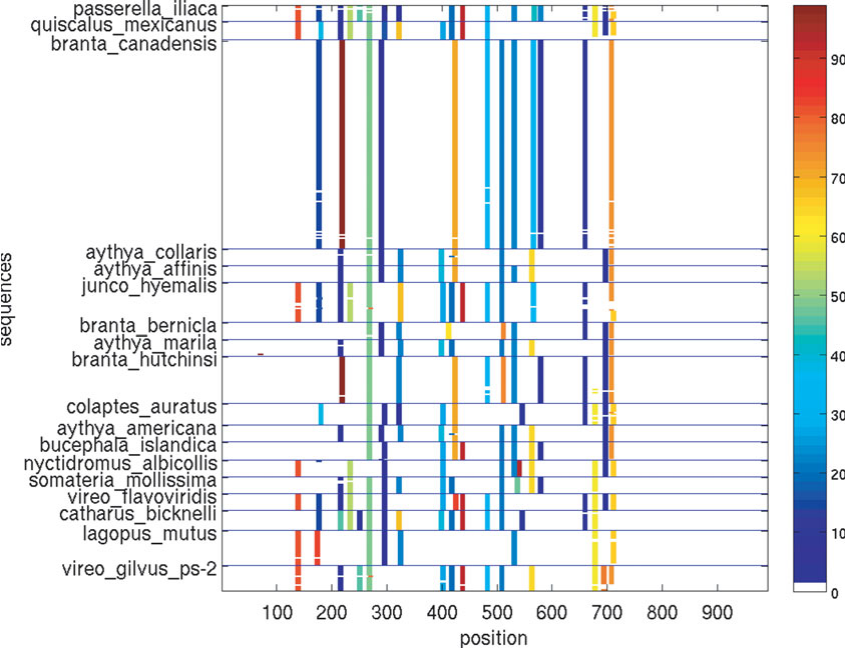
**Species sequence map for Birds of North America data set (top 100 spectrum (*k *= 10) features)**.

**Figure 9:**
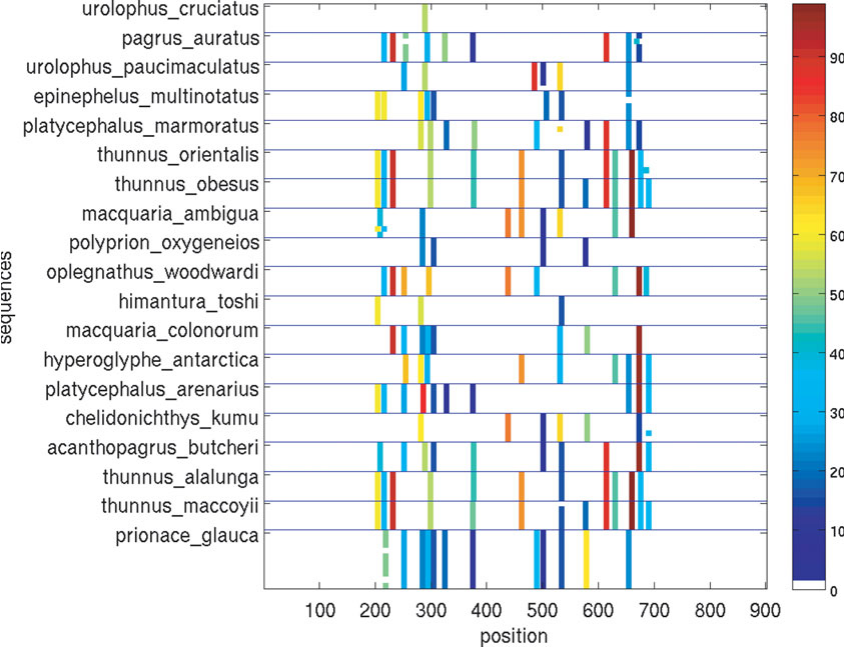
**Species sequence map for Fish of Australia data set (top 100 spectrum (*k *= 10) features)**.

**Figure 10:**
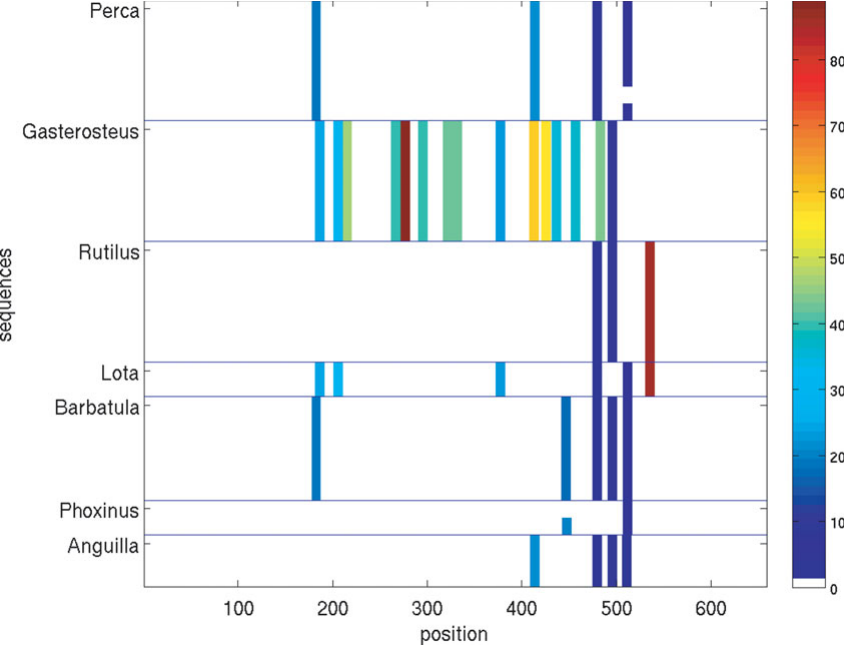
**Species sequence map for Fish larvae data set (top 100 spectrum (*k *= 10) features)**.

Figures 5, 6, 7, 8, 9, 10 show, for various barcode sets, the sequence-marker location maps for the top-100 spectrum features (highlighted in different colors). These marker location maps display how the identified top features are positioned within the barcodes. In the figures, the vertical axis corresponds to individual barcode sequences and the horizontal axis corresponds to the position within the aligned (for visualization purpose only) sequences. The blue horizontal lines in the figures indicate species class boundaries, while the color bars next to the maps indicate the color-to-marker index correspondences. For completeness Tables 7, 8, 9, 10, 11, 12 show the corresponding top-100 spectrum markers, ranked by the weights assigned by the feature selection algorithm.

**Table 7:** Top-100 spectrum features for Hesperiidae data set

**rank**	**feature**	**rank**	**feature**	**rank**	**feature**	**rank**	**feature**
1	TTATTATTAT	26	ATTAATATAC	51	TTTTTATAGT	76	GGAGCCCCTG
2	ATTATTATTA	27	CTTTCCCCCG	52	TTTTTTATAG	77	TATTAATTTC
3	TATTACCCCC	28	GCTTTCCCCC	53	ATTAATTTCA	78	AATATTGCTC
4	CCCCCTCTTT	29	TTAATATACG	54	TAATATACGA	79	TTTTTGATCC
5	AATTTTATTA	30	TATTATAATT	55	GTTTATCCCC	80	TTTTTTGATC
6	AGGAGCTATT	31	AGCTTTCCCC	56	TTTTTTTATA	81	ATATTGCTCA
7	ATTGCCCATC	32	GCTCCTGATA	57	CCTTCTTTAA	82	GAGCCCCTGA
8	TTGCCCATCA	33	TAATTTTATT	58	TGGAGATGAT	83	ATTGCTCATC
9	TTAGGAGCTC	34	CTCCTGATAT	59	TTATTACCCC	84	TGCTCATCAA
10	TCAAATACCT	35	TCCTGATATA	60	TTTATCCCCC	85	TTGCTCATCA
11	ACCTTTATTT	36	CTAATATTGC	61	CCCCTCTTTC	86	ATTATTAATT
12	TAGGAGCTCC	37	TAGGAGCCCC	62	ATTTAGCAAT	87	CTCATCAAGG
13	ATTTTATTAC	38	TGATCAAATA	63	GATTTAGCAA	88	GCTCATCAAG
14	GGAGCTATTA	39	TTAATTTTAT	64	TATTATTATT	89	TACCCCCCTC
15	ATTAGGAGCT	40	TTTGATCAAA	65	AGGAGCTCCT	90	TAGCTTTCCC
16	TATTGCCCAT	41	TTGATCAAAT	66	AATATTGCCC	91	ATATTAGGAG
17	CAAATACCTT	42	CTTTATTTGT	67	ATATTGCCCA	92	TATTAGGAGC
18	TGCCCATCAA	43	CCTTTATTTG	68	ATTATTAATA	93	TACTATTGTT
19	AATACCTTTA	44	TATTAATATA	69	ATTATTACCC	94	ATAGCTTTCC
20	AAATACCTTT	45	ATTAATTTTA	70	TTATTAATAT	95	CAATTATTAA
21	CCCCTGATAT	46	TACCTTTATT	71	AGATGATCAA	96	ACAATTATTA
22	GCCCCTGATA	47	AATTTTTTCT	72	GAGATGATCA	97	TTTAGCAATT
23	CCCATCAAGG	48	TATAGCTTTC	73	GGAGATGATC	98	CCCCCGAATA
24	CCCTGATATA	49	AGCCCCTGAT	74	TTTTATAGTT	99	TCCCCCGAAT
25	GCCCATCAAG	50	ATACCTTTAT	75	TTACCCCCCT	100	TTCCCCCGAA

**Table 8:** Top-100 spectrum features for Astraptes data set

**rank**	**feature**	**rank**	**feature**	**rank**	**feature**	**rank**	**feature**
1	TATACCAACA	26	CTTATATCAA	51	AATGGAGCTG	76	GGAGGAGACC
2	TTATACCAAC	27	TATATCAACA	52	ATGGAGCTGG	77	ATCTTGCCGG
3	TGAAAATGGA	28	TATCAACACT	53	GAAAATGGAG	78	CATCTTGCCG
4	AACTTCTTTA	29	TCAACACTTA	54	AATATACGAA	79	TCATCTTGCC
5	ACTTCTTTAA	30	TCTTATATCA	55	ATATACGAAT	80	CCGGTATTTC
6	CTTCTTTAAG	31	TTATATCAAC	56	ATTAATATAC	81	CGGTATTTCA
7	CTTTAAGATT	32	ACCCCCATCT	57	TAATATACGA	82	CTTGCCGGTA
8	TCTTTAAGAT	33	ATTACCCCCA	58	TATACGAATT	83	GCCGGTATTT
9	TTCTTTAAGA	34	ATTATTACCC	59	TATTAATATA	84	TCTTGCCGGT
10	TTTAAGATTA	35	TACCCCCATC	60	TTAATATACG	85	TGCCGGTATT
11	GAACTTCTTT	36	TATTACCCCC	61	AAAATGGGGC	86	TTGCCGGTAT
12	GGAACTTCTT	37	TTACCCCCAT	62	AAATGGGGCT	87	ATACGAATTA
13	TGGAACTTCT	38	TTATTACCCC	63	AATGGGGCTG	88	TACGAATTAA
14	AATCTTATAC	39	AATAATAGGT	64	ATGGGGCTGG	89	ACGAATTAAT
15	ACCAACACTT	40	AATAGGTGCC	65	GAAAATGGGG	90	AATATGCGAA
16	ATACCAACAC	41	AGGTGCCCCA	66	GGCTGGTACA	91	ATATGCGAAT
17	ATCTTATACC	42	ATAGGTGCCC	67	GGGCTGGTAC	92	ATGCGAATTA
18	CCAACACTTA	43	GGTGCCCCAG	68	GGGGCTGGTA	93	ATTAATATGC
19	CTTATACCAA	44	GTGCCCCAGA	69	TGAAAATGGG	94	GCGAATTAAT
20	TACCAACACT	45	TAGGTGCCCC	70	TGGGGCTGGT	95	TAATATGCGA
21	TCTTATACCA	46	TTGATTATTA	71	AGGAGACCCA	96	TATGCGAATT
22	AATCTTATAT	47	TGGAGGATTT	72	AGGAGGAGAC	97	TATTAATATG
23	ATATCAACAC	48	TGCCCCAGAT	73	GAGACCCAAT	98	TGCGAATTAA
24	ATCAACACTT	49	AAAATGGAGC	74	GAGGAGACCC	99	TTAATATGCG
25	ATCTTATATC	50	AAATGGAGCT	75	GGAGACCCAA	100	AATTGGAGGA

**Table 9:** Top-100 spectrum features for Bats of Guyana data set

**rank**	**feature**	**rank**	**feature**	**rank**	**feature**	**rank**	**feature**
1	ATAATTGGAG	26	AGCTTCTGAC	51	TTTCCCCGAA	76	GTAACAGCCC
2	TTGTAATAAT	27	CCTGTCCTAG	52	GTCTTATTAC	77	TAACAGCCCA
3	TTTGTAATAA	28	CTGTCCTAGC	53	TCTTATTACT	78	TGTTCTAGCA
4	ATAAGCTTCT	29	GCTTCTGACT	54	AGCCCATGCC	79	ATCACTATAC
5	TAAGCTTCTG	30	GTTCTAGCAG	55	TTATTACTAC	80	TCACTATACT
6	GTCCTAGCAG	31	TGTCCTAGCA	56	CCCATGCCTT	81	AAGCAGGAGT
7	TCCTAGCAGC	32	TTCTAGCAGC	57	GCCCATGCCT	82	CACTATACTA
8	CAACACTTAT	33	TCCTGTCCTA	58	TTATAATTGG	83	ACACTTATTC
9	CCTAGCAGCA	34	ATAGTAGGCA	59	ATTATAATTG	84	CCTAGCAGGC
10	AATATAAGCT	35	GTAACAGCTC	60	TATAATTGGA	85	AAACCTTAAT
11	ATATAAGCTT	36	TAACAGCTCA	61	AACAGCCCAT	86	AACCTTAATA
12	CTTCCTGTCC	37	AACAGCTCAT	62	TACCTATTAT	87	ACCTTAATAC
13	TTCCTGTCCT	38	ATCATAATTG	63	CCTGTTCTAG	88	TATTAGGTGA
14	AAGCTTCTGA	39	ATCAACACTT	64	CTGTTCTAGC	89	CCCCGAATAA
15	TCTTCCTGTT	40	TATCAACACT	65	TATTAATATA	90	CCCGAATAAA
16	ACAGCTCATG	41	TCAACACTTA	66	TTTATTACTA	91	TCTGACTCCT
17	CAGCTCATGC	42	TCATAATTGG	67	ATCAAACACC	92	TTCTGACTCC
18	AACACTTATT	43	ATATCAAACA	68	ATTAGGTGAT	93	TTTTATTACT
19	CTTCTGACTC	44	GAAGCAGGAG	69	CCTTTGTAAT	94	AGGTATCACT
20	TAGTAGGCAC	45	CTTCCTGTTC	70	GCCTTTGTAA	95	CTCAATATCA
21	ACAGCCCATG	46	TAATTGGAGG	71	TATCAAACAC	96	GGTATCACTA
22	CAGCCCATGC	47	TTCCTGTTCT	72	TCCTATTACT	97	GTAATAATTT
23	AATATAAAAC	48	AGTAGGCACT	73	CATAATTGGA	98	GTATCACTAT
24	ATATAAAACC	49	TCCCCGAATA	74	GAGCTATTAA	99	TAATAATTTT
25	TCTAGCAGCA	50	TTCCCCGAAT	75	GGAGCTATTA	100	TCAATATCAA

**Table 10:** Top-100 spectrum features for Birds of North America data set

**rank**	**feature**	**rank**	**feature**	**rank**	**feature**	**rank**	**feature**
1	ATCACAATAC	26	ACTTCATCAC	51	AAACAACATA	76	GATTCTTTGG
2	ATAATCGGAG	27	AACCTAGCCC	52	ACAACATAAG	77	TATACCAACA
3	ACCAACACCT	28	AACTTCATCA	53	ATTCTTCGAC	78	TAGCATTCCC
4	TACCAACACC	29	ACCTAGCCCA	54	AACTGACTAG	79	ATAGCATTCC
5	AAGCTTCTGA	30	ATCAACATAA	55	ACTGACTAGT	80	CGGAGCCTCA
6	AACATAAGCT	31	ATCAACTTCA	56	AACAACATAA	81	AGACGACCAA
7	ACATAAGCTT	32	TCAACTTCAT	57	AGCAATCAAC	82	CATGCCTTCG
8	CATAAGCTTC	33	ATACCAAACC	58	CAACTTCATC	83	GTAGACCTAG
9	TCCTACTCCT	34	CTAATCACTG	59	GGAGGAGACC	84	TAGACCTAGC
10	CCCCTATTCG	35	CTCACAATAC	60	CTCTCACAAT	85	ACCCCCCTAT
11	TTCTTCGACC	36	ATAAGCTTCT	61	ACAATACCAA	86	CCCCCCTATT
12	CCCTATTCGT	37	TAAGCTTCTG	62	ACGCCGGAGC	87	AACCCCCCTA
13	TCTTCGACCC	38	TCGTAATAAT	63	CACGCCGGAG	88	ATGCCTTCGT
14	GCCTTCGTAA	39	CAACATAAGC	64	GTCCTAATCA	89	TCATCACAAC
15	CCTTCGTAAT	40	GCAACCTAGC	65	TCCTAATCAC	90	TTCATCACAA
16	CTTCGTAATA	41	GGCAACCTAG	66	TGATTCTTTG	91	AAACTGACTA
17	AGCTTCTGAC	42	TAATCACTGC	67	TCCTCCTCCT	92	ATCTTCTCCC
18	GCTTCTGACT	43	AATACCAAAC	68	GAGGAGACCC	93	TCTTCTCCCT
19	GAGCCTCAGT	44	CAATACCAAA	69	ACATAGCATT	94	AAACCCCCCT
20	GGAGCCTCAG	45	ATAATTGGAG	70	GACATAGCAT	95	CAAACCCCCC
21	CAACATAAAA	46	TTCGTAATAA	71	CAGTAGACCT	96	AACCTAAACA
22	TCACAATACC	47	ACCAAACCCC	72	TCAGTAGACC	97	ACCTAAACAC
23	TCCTCCTACT	48	TACCAAACCC	73	TTCTGATTCT	98	CGTAATAATC
24	CACAATACCA	49	CATAGCATTC	74	TCTGATTCTT	99	ATCGGAGGAT
25	TCAACATAAA	50	TCTCACAATA	75	CATAAAACCC	100	TAATCTTCTT

**Table 11:** Top-100 spectrum features for Fish of Australia data set

**rank**	**feature**	**rank**	**feature**	**rank**	**feature**	**rank**	**feature**
1	AACATAAAAC	26	GACTTCTTCC	51	ACAGTCTACC	76	ATCTTCTCCC
2	ACATAAAACC	27	TAATAATTGG	52	CAGTCTACCC	77	TCTTCTCCCT
3	ATTATTAACA	28	AACATAAGCT	53	TAAATAATAT	78	ACTATTATTA
4	TTATTAACAT	29	ACATAAGCTT	54	AGCTTCTGAC	79	TATTATTAAC
5	TAACATAAAA	30	AATATCAAAC	55	CATAAAACCC	80	ATAGTAATAC
6	ATTAACATAA	31	CAATATCAAA	56	CCCCGAATAA	81	AGGAGACCCA
7	TTAACATAAA	32	TTATGATTGG	57	CCCGAATAAA	82	CTATTATTAA
8	TCCTTCTCCT	33	ACCAACACCT	58	GCTTCTGACT	83	TAATATAAAA
9	ATTATTAATA	34	TACCAACACC	59	TAGTAATACC	84	TTAATATAAA
10	TTATTAATAT	35	TTATTACAAC	60	TCATGATTGG	85	TTGACCCTGC
11	TATTAACATA	36	GAGACCCAAT	61	CCTCGAATAA	86	AATAAACAAC
12	GAACAGTTTA	37	GGAGACCCAA	62	CTCGAATAAA	87	AATTTTATTA
13	TGAACAGTTT	38	TTTATTACAA	63	CTTCTTCTCC	88	ATTACAATGC
14	GAGGAGACCC	39	ATACCAATTA	64	AATACCAAAC	89	ATTTTATTAC
15	GGAGGAGACC	40	CTTTACCAAC	65	CAATACCAAA	90	TTACAATGCT
16	TGACTTCTTC	41	GGAGGAGGAG	66	GAGGAGGAGA	91	TTGGAAACTG
17	ATCAAACACC	42	TACCAATTAT	67	ATGAGCTTCT	92	TTTGACCCTG
18	TATCAAACAC	43	TTTACCAACA	68	ATGATTGGAG	93	TTTGGAAACT
19	TTCTTCTCCT	44	AACAGTCTAC	69	TGAGCTTCTG	94	AATAAATAAT
20	AATATAAAAC	45	ACAGACCGAA	70	TTATGATCGG	95	ATTAATATAA
21	ATATAAAACC	46	CAGACCGAAA	71	ATAAATAATA	96	GAGGGGACCC
22	CGAATAAATA	47	GAACAGTCTA	72	TTACCAACAC	97	GGAGGGGACC
23	GAATAAATAA	48	TGAACAGTCT	73	TTTCCTCAAT	98	AATATGAGCT
24	TCTTTGACCC	49	ATAATTGGTG	74	ATATCAAACA	99	ACCCTGCAGG
25	TTCTTTGACC	50	TCCTTCTTCT	75	ATAATTGGAG	100	AGACCGAAAC

**Table 12:** Top-100 spectrum features for Fish larvae data set

**rank**	**feature**	**rank**	**feature**	**rank**	**feature**	**rank**	**feature**
1	CGCAATCCTC	26	CCAGTCAATG	51	AATAAAGGAT	76	AGTGGATCAT
2	GCAATCCTCT	27	CCCATGTGGA	52	AATAAATAAC	77	AGTTACAACT
3	GCGCAATCCT	28	CCCCATGTGG	53	AATAACCCCC	78	ATAAACAGAA
4	ATCAACGAAC	29	CCCCCATGTG	54	AATGACCCTA	79	ATAAAGGATT
5	CGCAATCCCC	30	CCCCGTGCAG	55	AATTGATCTC	80	ATAAATAACC
6	GATCAACGAA	31	CTCCCCGTGC	56	ACAACTCTAA	81	ATAACCCCCA
7	GCAATCCCCT	32	TCCAGTCAAT	57	ACAAGATGGA	82	ATCAACGGAC
8	GCGCAATCCC	33	TCCCCGTGCA	58	ACACTAAAGT	83	ATCATGTCAA
9	TCAACGAACC	34	TGACCAAAAA	59	ACAGCTGAGA	84	ATCCTCTTTT
10	ACCCTAGGGA	35	AAAAGATCCG	60	ACCCACCCTG	85	ATCGACGAGG
11	AGTTACCCTA	36	AAACAGAATT	61	ACCCCTCCTA	86	ATCTCCCCGT
12	CCCTAGGGAT	37	AAAGATCCGG	62	ACCCTGATGT	87	ATGACCCTAA
13	CCTAGGGATA	38	AAAGGATTGA	63	ACCTAGTTAC	88	ATGGAACCCA
14	CGATCAACGA	39	AAAGTGGATC	64	ACGGACCTAG	89	ATGTCAATGA
15	GTTACCCTAG	40	AAATAAAGGA	65	ACTAAAGTGG	90	ATGTGGAATG
16	TACCCTAGGG	41	AAATAACCCC	66	ACTCTAATAA	91	ATTGAACAAG
17	TTACCCTAGG	42	AACAAGATGG	67	AGAAGCGGGG	92	ATTGATCTCC
18	TCTGACCAAT	43	AACCCACCCT	68	AGACACTAAA	93	CAACGGACCT
19	TTTCAAGTCA	44	AACGGACCTA	69	AGAGTCCATA	94	CAACTCTAAT
20	CTGACCAAAA	45	AACTCTAATA	70	AGAGTTACAA	95	CAAGATGGAA
21	TCTGACCAAA	46	AAGATGGAAC	71	AGATGGAACC	96	CAATCCCCTC
22	AAACTAAGAG	47	AAGCGGGGAT	72	AGCGGGGATT	97	CAATCCTCTT
23	AACCCCCATG	48	AAGGATTGAA	73	AGCTGAGAGT	98	CAATGACCCT
24	ACCCCCATGT	49	AAGTGGATCA	74	AGGATTGAAC	99	CACCCCTCCT
25	CAGTCAATGA	50	AATAAACAGA	75	AGTCCATATC	100	CACCCTGATG

The results indicate that different species classes have distinct distributions (in terms of both spectrum marker types and their locations) of the first few spectrum markers (top 100 shown). This allows one to accurately distinguish species between each other by solely using the marker sets. As a consequence, these small sets of features can serve as signatures for efficient and accurate classification and identification, among the set of considered species.

### New species detection

In the classification task that we considered in previous sections, a species label for a new barcode is predicted according to the class (species) models learned from training examples. In this section we consider the problem of identifying whether a barcode belongs to a species in the training set or to a new, unknown species.

We simulate the new species detection by holding out barcode samples from one of the species in the barcode dataset. Barcode samples belonging to this species are then presented to the model that only contains barcodes from the remaining "known" species. The query barcode is labeled as a new species if its distance to the nearest barcode in the "known species" set exceeds the average distance between barcodes in the class of that closest known species barcode. Otherwise, the query barcode is assigned to the class of the closest known species. We then measure the new species detection error rate by holding out each species in the dataset and averaging the individual species error rates. The average new species detection error rates are reported in Table 13. The error rates in Table 13 are, thus, the errors from assigning a sample from held-out classes (i.e. new species) to the existing train classes (known species). Table 14 shows new species detection error rates in a slightly more comprehensive but also more realistic task, where we held-out not only the barcodes from the "unknown" species but also some random barcodes from the known species. Hence, the error rates in Table 14 include errors from incorrectly assigning the "new species" label to the barcode samples from one of the known classes but also the errors from assigning the query barcode from new species to one of the existing classes.

**Table 13:** New species detection (average per-class error, %)

**Dataset**	**error**	**# classes with 0% error**
ACG	14.12	474/573
Hesperiidae	17.53	288/364
Astraptes	7.81	10/12
Bats of Guyana	5.80	90/96
Birds of North America	16.36	524/656
Fish of Australia	16.21	174/211

**Table 14:** New species detection (average error rate, %)

**Dataset**	**Error**
ACG	10.29
Hesperiidae	10.88
Astraptes	8.47
Bats of Guyana	9.95
Birds of North America	15.54
Fish of Australia	14.92
Fish larvae	15.77

**Figure 11:**
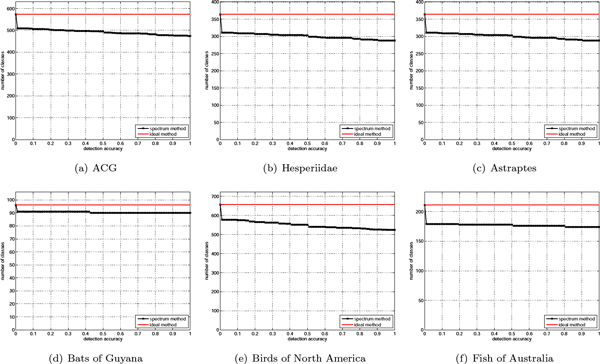
**New species detection performance**.

As we can see from the results, using the nearest neighbor approach results in about 85-90% correct identification of the samples belonging to new species. To further investigate the errors committed here we show a slightly more informative representation of the new species detection results in Figure 11. The black curve in each panel indicates the number of classes (vertical axis) with the detection accuracy equal to or higher than the corresponding value on the horizontal axis, for each of the barcode datasets. For example, in the ACG set there are about 480 species that can be predicted with the accuracy of 90% or higher. The performance of an ideal method would result in the horizontal solid-red line. In all cases, performance of the spectrum method relatively closely follows the ideal method. Majority of incorrect assignments occur on very few species, which is evident from the sudden initial drops in the curves. We observe that incorrect assignments of the samples from new species to the existing classes often resulted from the presence of the (nearly) duplicate sequences in the training set. For instance, in the Astraptes dataset, query sequences from SENNOV were closest to an identical sequence which is also present in the YESENN species. This points to the need for alternative sources of information in cases where the barcodes may not be sufficient to discriminate among species but also to the need for additional curation in some of the datasets.

### Barcode clustering

In this section, we consider the problem of arranging barcode sequences into groups (clusters) to establish relationships between sequences and automatically discover class structure. Clustering can be useful for putative samples with no prior evidence of species assignments. Clustering-based evaluation also allows one to eliminate the influence of the classification training (weight estimation) process, which is an additional factor that affects the classification performance. As a consequence, clustering may provide a more direct insight into the quality of the similarity measure alone.

To evaluate the results of clustering we compare the partitioning obtained by the clustering model with the partitioning given by the known species membership. We perform clustering experiments using the recently proposed affinity propagation algorithm [39] which makes use of the computed similarity scores. We report clustering results using the alignment-free methods only in Table 15, in the light of the classification results which we presented earlier across different similarity metrics. As we can see from the table, clustering of barcode datasets results in accurate partitioning of the barcodes into groups that are similar to the true species-induced classes. We use Rand [40] and Jaccard indices to evaluate the clustering quality. Rand = (*a *+ *d*)/*T*, Jaccard = *a*/(*a *+ *b *+ *c*), where *a *is the number pairs with the same class label assigned to the same cluster, *b *is the number of points with the same class label assigned to different clusters, *c *is the number of pairs with different class labels placed into the same cluster, *d *is the number of pairs with different labels placed into different clusters, and *T *is the total number of pairs.

**Table 15:** Clustering results (using spectrum similarity measure)

**Dataset**	**#clusters**	**error, %**	**Rand index**	**Jaccard index**
ACG	644	2.84	99.85	83.96
Hesperiidae	382	4.44	99.79	86.42
Astraptes	17	1.51	95.59	81.59
Bats	98	0.95	99.21	86.58
Birds	650	5.25	99.90	86.59
Fish Australia	235	2.52	99.94	93.07
Fish larvae	7	2.86	98.66	95.51

**Figure 12:**
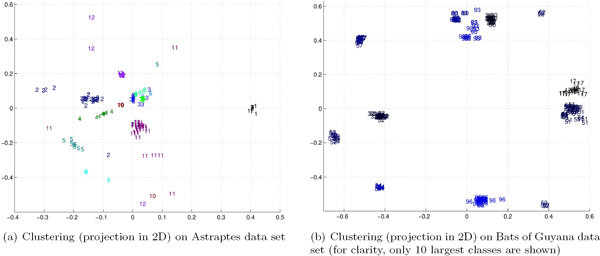
**Clustering results for barcode sequences using alignment-free spectrum method (data is projected onto 2D plane)**.

To further illustrate the clustering results, we show in Figure 12 projections of the barcode data onto a 2D plane. We note the agreement of species labels and proximities in the embedded space. This suggests possibility of using clustering with alignment-free methods as an efficient and accurate tool for exploratory analysis of newly obtained barcodes.

### Experimental running time analysis

Computational complexity of evaluating similarity on large sets of barcodes may be a prominent factor in practical barcoding applications. We evaluate the computational speed of different methods by measuring running time for computing similarity scores among all pairs of sequences in a barcode dataset. The running times are obtained by running the optimized versions of all targeted methods on a single 3.0 GHz quad core CPU using 2 GB RAM (Dell PowerEdge 2950). Table 16 compares running times for the *alignment-free *kernel methods as well as PSI-BLAST and alignment-based (Smith-Waterman) methods. As we can see from the table, alignment-free kernel methods are significantly faster compared to computationally demanding alignment-based (Smith-Waterman) methods. For example, it takes about 60 seconds to evaluate 4267-by-4267 similarity matrix using the spectrum method compared to 64800 seconds (18 h) for computing the Smith-Waterman scoring matrix. These results are not surprising given the complexity analysis mentioned in Section 'Methods' and further studied in [31]. The low computational complexity of the alignment-free scores, besides its appeal for analytics on large datasets, also opens the possibility for using these metrics on conceptualized handheld barcode scanners that may be designed in the future.

**Table 16:** Running time (kernel computation), s

**Dataset**	**PSI-BLAST**	**Smith-Waterman**	**spectrum (k = 10)**	**mismatch**
ACG	2557	64818	60.328	366.39
Hesperiidae	891	19378	16.16	90.26
Astraptes	99	854	0.89	2.15
Bats	239	2818	1.91	12.12
Birds	1147	27311	18.75	149.67
Fish Australia	203	3424	1.57	9.95
Fish larvae	1.9	39.58	0.23	0.16

## Conclusion

In this work we demonstrate that newly developed alignment-free methods can serve as efficient and accurate analytical tools for DNA barcoding problems. The new alignment-free methods provide highly accurate and computationally efficient identification and classification of barcode sequences as we show on a set of various barcode collections. Using new alignment-free scoring approaches demonstrates excellent performance in comparison with more computationally demanding, traditional alignment-based methods. The use of alignment-free scoring methods allows discovery of natural groups (clusters) in barcode collections that accurately reflect the species-based groupings. This reflects potentially high agreement between the proposed fragment-induced sequence similarity measures and the within and across species barcode diversity. Finally, we show that the spectral methods also foster discovery of within-barcode markers that point to critical differences among barcodes of different sample groups. These markers can serve both as the sparse and robust barcode codes and as possible pointers to within barcode loci that deserve further investigation. Our experiments finally suggest that it may be possible to further improve the performance of our spectral scores by merging them with the position-based metrics, such as the Kimura distance. Currently, the spectral kernels rely on the 0/1 scoring within each fragment. While implementing Kimura scoring within *k*-mers is possible, a direct implementation adversely affects the spectral algorithm's efficiency, with the complexity becoming quadratic in the sequence length. This leaves open an avenue for future research into efficient spectral barcoding algorithms for arbitrary fragment scores.

## Competing interests

The authors declare that they have no competing interests.

## Authors' contributions

All authors contributed equally to this work.
